# Association between Pathogenic Variants of Diarrheagenic* Escherichia coli* and Growth in Children under 5 Years of Age in the Global Enteric Multicenter Study

**DOI:** 10.4269/ajtmh.22-0096

**Published:** 2022-06-06

**Authors:** Rina Das, Parag Palit, Md Ahshanul Haque, Tahmeed Ahmed, A. S. G Faruque

**Affiliations:** Nutrition and Clinical Services Division, International Center for Diarrheal Disease Research, Bangladesh (icddr,b), Dhaka, Bangladesh

## Abstract

There is a lack of information highlighting associations between different pathogenic variants of diarrheagenic *Escherichia coli* and childhood growth. Pathogenic variants of *E. coli* from stool samples, collected from 22,567 children enrolled in the Global Enteric Multicenter Study from December 2007 to March 2011, were detected by real-time polymerase chain reaction. We estimated the associations of different pathogenic variants of diarrheagenic *E. coli* with child growth. The association between an explanatory variable and the outcome variable was assessed using multiple linear regression, where the dependent variables were height-for-age, weight-for-age, and weight-for-height *z*-scores, and the independent variable was the presence of different pathogenic variants of diarrheagenic *E. coli*. After adjusting for potential covariates, such as age, gender, diarrhea, breastfeeding status, mother’s education, number of under-5 children, handwashing practice, handwashing material, source of drinking water, wealth index, available toilet facility, copathogens, comorbidity, time, and study site, the multivariable model identified a negative association between different pathogenic variants of diarrheagenic *E. coli* and child growth. Our analyses may provide the cornerstone for prospective epidemiologic investigation for the development of preventive measures for diarrheagenic *E. coli* and combat childhood undernutrition.

## INTRODUCTION

Childhood malnutrition is linked to a higher risk of death from diarrhea, pneumonia, and other infectious diseases, as well as growth failure, cognitive delay, and productivity loss.^1^^,^^2^ Diarrhoeagenic *Escherichia coli* causes the most common bacterial gastrointestinal infection in developing nations, especially among infants and young children aged under 2 years.^3^^–^^7^

In developing nations, acute diarrhea is still the most common cause of infantile diarrhea and the most common attributing factor for malnutrition.^7^ Several variants of *E. coli* constitute the nonpathogenic gut microbiome. However, some variants of *E. coli* have evolved with the capacity to exert virulence causing different manifestations of gastrointestinal illness and account for the most common cause of childhood diarrhea.^8^ Traveler’s diarrhea (enterotoxigenic *E. coli* or ETEC), chronic diarrhea (enteroaggregative *E. coli* or EAEC), and watery diarrhea in children are all clinical symptoms associated with diarrheagenic *E. coli* infection (enteropathogenic *E. coli* or EPEC).^8^^,^^9^

Evidence suggests that several enteropathogens, including EAEC, have been linked to childhood malnutrition.^10^^–^^12^ Since its discovery by patterns of adherence to HEp-2 (human epithelial type 2) cells in *E. coli* isolates from Chilean children with diarrhea,^13^ EAEC has become increasingly recognized as a major enteropathogen. A wide array of interacting features that reside on both the chromosome and the plasmid have been reported to mediate the genetic determinants and biological processes for EAEC pathogenicity.^14^ EAEC is diverse in terms of genetic content as previously described.^15^ The *eae* serotype of EAEC exhibited positive relationships with poor linear development at 24 months of age in the MAL-ED study (Malnutrition and Enteric Disease Study).^16^

A cross-sectional case-control study involving Brazilian children aged 2–36 months found a stronger link between tEPEC (typical EPEC) infections and clinical severity of diarrhea and undernutrition, compared with the cases of aEPEC (atypical EPEC) infections.^17^ On the basis of the presence of the *Escherichia coli* adherence factor (EAF) plasmid encoding bundle-forming pili (BFP), EPEC can be subgrouped into tEPEC and aEPEC.^18^ Typical EPEC is usually linked to incidences of gastroenteritis, even severe diarrhea among infants, whereas aEPEC is associated with a wide array of clinical manifestations, ranging from asymptomatic colonization to prolonged diarrhea, based on different settings.^8^^,^^18^^,^^19^ Findings from studies carried out across 13 developing countries showed that isolates of aEPEC accounted for 78% of all EPEC-associated diarrheal cases among children aged less than 5 years.^20^ Additionally, EPEC may lead to severe nutrient malabsorption, resulting in nutritional consequences and eventual persistence of diarrhea.^21^

Recent findings have yielded a better understanding of the potential consequences of enteric infections beyond symptomatic diarrhea, with ETEC being one of the most common causes of diarrhea among children in underdeveloped countries and a significant contributor to childhood stunting.^22^ Human ETEC strains can produce a heat-labile enterotoxin (LT) that resembles cholera toxin and one or more heat-stable enterotoxins (ST) including human ST (STh) or porcine ST (STp). Strains can produce both LT and ST (LT/ST) or be ST-only or LT-only. Most ETEC encodes colonization factors that allow the pathogen to attach to proximal small intestine enterocytes, the critical site of host–parasite interaction, before expressing enterotoxins that decrease villus tip cell absorption and evoke secretion of electrolytes and water by crypt cells.^23^

Diarrheagenic *E. coli* have developed different strategies for the establishment of infection in their host. Understanding these pathogenic mechanisms has led to the development of a specific vaccine against *E. coli* strains into different pathotypes.^24^ In most cases, this genetic information has been horizontally acquired and belongs to the flexible *E. coli* genome, such as plasmids, bacteriophages, and genomic islands. These genomic regions contribute to the rapid evolution of *E. coli* variants as they are frequently subjected to rearrangements, excision, and transfer as well as the further acquisition of additional DNA thus contributing to the creation of new (pathogenic) variants.^25^ The genetic diversity of *E. coli* has been underestimated. Our analyses may lay the cornerstone for a prospective epidemiologic investigation for a potential vaccine development aimed at reducing the burden of *E. coli* infections and combating childhood malnutrition.

However, a complete understanding of the epidemiology of the pathogenic variants of *E. coli* and their relationship with childhood growth is still lacking. In the current study, we hypothesized that diarrhoeagenic *E. coli* is responsible for child undernutrition among those under 5 years of age. We used the GEMS (Global Enteric Multicenter Study) data set derived from four countries of sub-Saharan Africa and three countries from South Asia, where *E. coli* was detected from a stool sample of the participants on enrollment and growth outcomes were evaluated at 60 days follow-up. Our study aimed to find out the possible association between different pathogenic variants of *E. coli* and consequent growth failure among under-5 children.

## MATERIALS AND METHODS

### Study site.

The study was conducted at seven different geographical locations, namely: The Gambia, Kenya, Mali, Mozambique, Bangladesh, India, and Pakistan. Details about the study sites are described elsewhere.^26^^,^^27^

### Study design and participants.

**Figure 1.**
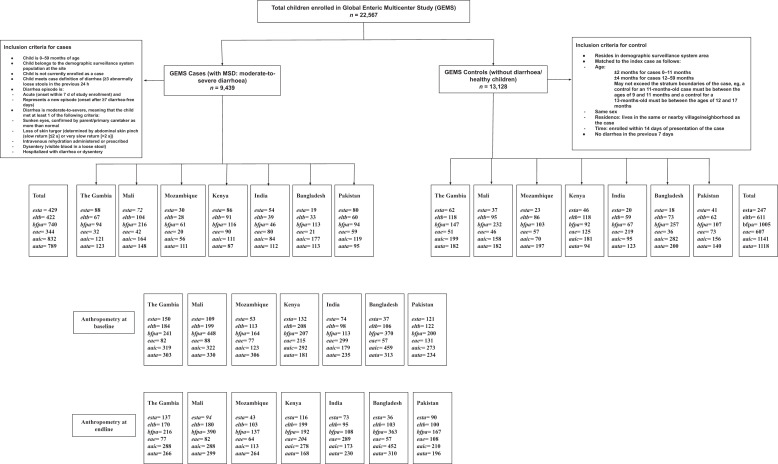
Study flow chart.

The design and methodology of the GEMS have been reported earlier.^27^ For this study, data were extracted from cases and controls enrolled in the GEMS, a 3-year (December 2007 to March 2011), prospective, age-stratified, matched case-control study of moderate-to-severe diarrhea (MSD) episodes^27^ among under-5 children, residing within a specified and enumerated population.^27^^,^^28^ Within 14 days of each enrolled case (who visited the health facility with MSD), one to three randomly selected age- and sex-matched community children (healthy children) were enrolled as controls from the same or neighborhood community.^29^ In our study, we used weighted means (ordinary arrhythmic mean, commonly used during descriptive statistics) of baseline and endline HAZ (height-for-age *z*-score), WAZ (weight-for-age *z*-score), and WHZ (weight-for-height *z*-score) from enrolment to follow-up for the different pathogenic variants of diarrheagenic *E. coli* (+) children enrolled in GEMS (Figure 1). The *est* (heat stable) and *elt* (heat-labile) serotypes were for ETEC; *bfpA* and *eae* serotypes were for EPEC; *aaiC* and *aatA* serotypes were for EAEC.

### Collection and processing of stool samples.

In the GEMS, stool samples were delivered to the laboratory in cooler boxes after collection. A separate fecal aliquot was placed in two tubes, one containing Cary–Blair media^30^ and the other buffered glycerol saline (BGS),^31^ either at the time of collection or after the samples were received in the laboratory. When a fecal specimen could not be collected, a rectal swab was obtained during enrollment and promptly placed into the tubes containing Cary–Blair and BGS medium. Study staff critically monitored the time between sample collection and transport to the laboratory, whereby the interval between stool collection and transport media inoculation did not exceed 6 hours, and the duration between placing the specimen in transport media and accession did not exceed 18 hours. For following examinations, separate aliquots of the stool samples were made and stored in −80°C freezer, before further analysis.

### Fecal microbiology.

The GEMS protocol incorporated traditional bacterial culture, largely to allow central laboratories to independently validate the growth of the involved pathogens and characterize them further for virulence, serologic, and antibiotic resistance features, as described elsewhere.^32^

### *Escherichia coli* isolation and identification.

*Escherichia coli* from the stool samples were isolated by characteristic microbiological and biochemical tests, as mentioned elsewhere, after that the *E. coli*-positive stool samples were assessed for the different pathogenic variants.

The different pathogenic variants of *E. coli*, namely ETEC, EPEC, and EAEC, were detected using a multiplex polymerase chain reaction (PCR), as previously published,^33^ modified for GEMS. Specific primers were used for the detection of different genomic variants of *E. coli* in the multiplex quantitative PCR (qPCR) panel. These primers were derived from the stable-enterotoxin-producing gene (*est*) for the detection of ETEC, gene for intimin outer membrane adhesion protein (*eae* gene) for the detection of aEPEC, bundle-forming pilus (BFP) of plasmid origin for the detection of tEPEC, and plasmid-encoded gene *aatA* for the detection of EAEC.

### Outcome variables.

The length or HAZ, WAZ, and WHZ were the key measures of growth in our analyses, with the average of three repeated measurements for each child at each visit (during enrollment and at 60 days follow-up) computed according to WHO recommendations.^27^ At the time of constructing the GEMS master data file for any analysis, implausible height values and values that were inconsistent between enrollment and follow-up^34^ were not considered. We used weighted means of baseline and endline HAZ, WAZ, and WHZ for the different pathogenic variants of diarrheagenic *E. coli* among the *E. coli*-positive enrolled as cases and as well as the *E. coli*-negative controls in GEMS from enrolment to follow-up in our analysis.

### Data collection.

The passing of three abnormally loose or watery stools per 24 hours was defined as diarrhea,^27^ whereas acute diarrhea was defined as having had 1–6 days of diarrhea in the previous 21 days. Many of the factors, such as vomiting (at least three times per day), fever (at least 38°C), and presence of obvious blood in the stools, were examined retrospectively (maternal recall).^27^ Both exclusively and partially breastfed children were referred to as “breastfed.” The information about the enrolled child’s family (defined as a group of people sharing a common cooking fire) included the mother as the major caregiver, mother’s education (illiterate or literate), household size (including the number of children under the age of 5), building materials (most common floor material: earth, sand, dung, and others), handwashing practices (before nursing or preparing baby food; after handling animals and cleaning a child), access and the main source of drinking water (tube well water and nontube well water), water treatment (water treatment method of drinking water available or not), improved toilet facilities (toilet facility for disposal of human fecal waste available or not), animals on the premises (sheep, goat, fowl, cow, dog, and cat), and the use of handwashing, were all the explanatory variables that were studied in this analysis. To analyze possible factors linked with disease, households were classified into socioeconomic status (SES) quintiles based on the wealth index quintiles (poor, lower-middle, middle, upper-middle, and richest).^27^ GEMS field staff visited each enrolled child’s household approximately 60 days after enrolment and detailed information of morbidity of the participant were recorded as published elsewhere.^27^

### Anthropometry.

Height, weight, and mid-upper arm circumference (MUAC) were measured at enrollment and the 60-day follow-up visit for each child, and details of measuring methods have been described elsewhere.^27^ Using a digital scale calibrated every day (model 314, Tanita Corp of America, Arlington Heights, IL), weight (to the nearest 0.1 kg) was measured following the standard guidelines for measurement of weight. In the recumbent position, the length of children 0–23 months of age or those who were older but unable to stand unassisted were measured using a board with a fixed head and sliding foot piece (to the nearest 0.1 cm) (Shorr Productions, Olney, MD). In children 2 years of age and older, the same apparatus was used to measure standing height. To calculate MUAC to the nearest 0.1 cm, a 25-cm paper single-slotted insertion tape was used (Shorr Productions).^35^ Length/height and MUAC were measured three times each and the average was estimated.^36^ Using the WHO Child Growth Standards as the reference population, the height/length-for-age, weight-for-age, and weight-for-height/length *z*-scores (HAZ, WAZ, and WHZ) were measured using a WHO SAS macro.^37^^,^^38^

### Statistical analysis.

We reported the child, maternal, and household-level characteristics by using mean and SD for continuous variables and frequency as a percentage for categorical variables to summarize the data. A paired *t* test was used to test statistical significance of an observed difference between baseline and endline (60 days’ follow-up after enrolment) *z*-score among these study children. To assess the association between presence of virulence-related genes of *E. coli* at baseline and the change in the child’s HAZ, WAZ, and WHZ in the subsequent 60 days, we used a generalized linear model, where the explanatory variable was presence of virulence-related genes of *E. coli* and the out-come variable was child anthropometry (HAZ, WAZ, and WHZ). The repeated measure was used as the time variable in STATA. The explanatory variable (presence of pathogenic variants of *E. coli*) was used individually in the generalized linear model at first to investigate its unadjusted impact on the outcome variable (WAZ, HAZ, and WHZ). All the factors, age, gender, diarrhea at enrolment, breastfeeding status, mother’s education, number of under-5 children at the house, handwashing before nursing a child and after cleaning a child, handwashing material, main source of drinking water, wealth index, available toilet facility, copathogens *Campylobacter* and *Giardia* which were mostly found, comorbidity (malaria, typhoid, pneumonia, diarrhea, and dysentery), time (anthropometry in two time points: on enrollment and day 60 follow up), and study site, suggesting the association with the outcome variable in the literature review were chosen for multivariable modeling. The variance inflation factor (VIF) was calculated to detect multicollinearity, and no variable with a VIF value greater than 5 was identified in the final model. We estimated the coefficient and its 95% CI to describe the precision of the point estimate. During the analysis, a *P* value of < 0.05 was considered statistically significant. STATA 15.0 IC (Stata Corp LLC, College Station, TX) was used to analyze all of the data.

### Ethical consideration.

Before the beginning of the research, the clinical procedure, consent forms, case report forms (CRFs), field procedures, and other supporting materials were approved by the ethics committees and the related scientific review boards at the University of Maryland School of Medicine and the committees overseeing each site and their coordinating partners from other institutions. The signed informed consent forms for the children’s inclusion in the study were collected from the children’s parents/guardians (both sick cases and healthy controls).

## RESULTS

### General characteristics of the study population.

A total of 10,924 *E. coli* (+) stool samples were collected from 22,567 enrolled participants who completed the follow-up 60 days after the enrollment. The stool samples collected from all the participants at the different study sites were assessed for the presence of different pathogenic variants of *E. coli* using quantitative real-time PCR (qPCR). The general characteristics of the study children are presented in Table 1. Among the enrolled children, 57% were male, around 40% of the children are from the 0–11 months age group. The mean and SD of MUAC, HAZ, WAZ, and WHZ were 14.08 ± 1.34, 1.34 ± 1.33, −1.26 ± 1.37, and −0.72 ± 1.48, respectively, at enrollment. About 70% of children were breastfed, 42% had MSD, and 10% had dysentery, and 27.26% dysentery cases were from Bangladesh. Maternal education was lowest (illiterate) in The Gambia (89.04%). In Bangladesh, 81% of enrolled children lived in a house where the floor was made of earth/sand. About 86% of enrolled children had an animal in their household. One-fourth of the households were users of a tube well water as the main source of drinking water and for domestic purposes. Except for Kenya improved toilet facility (toilet facility for disposal of human fecal waste available) was available in more than 90% of the houses; almost 75% used soap and water during handwashing. Handwashing practice was observed among participants around 40% of the time before nursing a child and 46.25% after cleaning the child. In Kenya, fewer mothers were illiterate (1.31%); in The Gambia, only 2.62% of the mother were the primary caretaker of the children; in Bangladesh, most of the children lived in a house where the floor was made of earth or sand; in Pakistan, site presence of animal at household was less observed (30%); almost all the household used tube well water as the main source of drinking water in Bangladesh; except Kenya, more than 90% participants used improved toilet facility; 55.11% households used soap during handwashing in India.

**Table 1:** General characteristics of GEMS children (*N* = 22,567) from Bangladesh, India, Pakistan, Gambia, Mali, Mozambique, and Kenya

	The Gambia, *N* = 2,598 (%)	Mali, *N* = 4,097 (%)	Mozambique, *N* = 1,976 (%)	Kenya, *N* = 3,359 (%)	India, *N* = 3,582 (%)	Bangladesh, *N* = 3,859 (%)	Pakistan, *N* = 3,096 (%)	Total *N* = 22,567(%)
Age (months)*	16.53 ± 9.63	19.02 ± 13.0	13.87 ± 10.52	18.10 ± 13.41	18.44 ± 13.06	18.77 ± 12.68	16.79 ± 12.65	17.71 ± 12.51
Age group (months)								
0–11	985 (37.9)	1,454 (35.5)	1,072 (54.2)	1,346 (40.1)	1,357 (37.9)	1,428 (37.0)	1,266 (40.9)	8,908 (39.5)
12–23	1,094 (42.1)	1,377 (33.6)	586 (29.6)	1,031 (30.7)	1,186 (33.1)	1,237 (32.1)	1,075 (34.7)	7,586 (33.6)
24–59	519 (19.9)	1,266 (30.9)	320 (16.2)	982 (29.2)	1,039 (29.0)	1,194 (30.9)	755 (24.4)	6,075 (30.9)
Gender (male)	1,426 (54.9)	2,264 (55.3)	1,196 (60.5)	1,903 (56.7)	2,031 (56.7)	2,237 (57.9)	1,766 (57.0)	12,823 (56.8)
Breastfeed	1,731 (66.6)	2,540 (62.0)	1,480 (74.9)	2,167 (64.5)	2,812 (78.5)	3,179 (82.4)	1,871 (60.4)	15,780 (69.9)
Anthropometry								
At enrollment								
WAZ*	–1.39 ± 1.45	–1.1 ± 1.34	–0.79 ± 1.5	–0.91 ± 1.29	–1.31 ± 1.25	–1.33 ± 1.16	–1.89 ± 1.41	–1.26 ± 1.37
HAZ*	–1.22 ± 1.41	–0.90 ± 1.28	–1.40 ± 1.40	–1.39 ± 1.29	–1.36 ± 1.20	–1.29 ± 1.12	–1.98 ± 1.48	–1.34 ± 1.33
WHZ*	–1.02 ± 1.73	–0.86 ± 1.54	0.024 ± 1.76	–0.19 ± 1.32	–0.77 ± 1.28	–0.86 ± 1.16	–1.08 ± 1.46	–0.72 ± 1.48
MUAC*	14.0 ± 0.3	14.2 ± 1.2	14.5 ± 1.5	14.3 ± 1.4	14.0 ± 1.3	14.3 ± 1.2	13.3 ± 1.4	14.1 ± 1.3
At 60 days after enrollment								
WAZ*	–1.30 ± 1.36	–0.99 ± 1.23	–0.65 ± 1.38	–0.90 ± 1.28	–1.28 ± 1.24	–1.28 ± 1.14	–1.91 ± 1.38	–1.20 ± 1.32
HAZ*	–1.36 ± 1.32	–0.95 ± 1.23	–1.51 ± 1.58	–1.56 ± 1.25	–1.52 ± 1.15	–1.42 ± 1.11	–2.13 ± 1.40	–1.46 ± 1.31
WHZ*	–0.83 ± 1.53	–0.67 ± 1.41	0.21 ± 1.55	–0.07 ± 1.28	–0.63 ± 1.28	–0.72 ± 1.12	–1.01 ± 1.45	–0.57 ± 1.40
MUAC*	14.4 ± 0.3	14.6 ± 1.2	14.9 ± 1.5	14.5 ± 1.4	14.3 ± 1.3	14.6 ± 1.2	13.6 ± 1.4	14.4 ± 1.3
Clinical features								
Diarrhea	1,029 (39.6)	2,033 (49.6)	682 (34.5)	1,476 (43.9)	1,568 (43.8)	1,394 (36.1)	1,258 (40.6)	9,440 (41.8)
Dysentery	220 (8.5)	243 (5.9)	123 (6.2)	166 (4.9)	188 (5.3)	1,052 (27.3)	246 (7.9)	2,238 (9.9)
fever	196 (7.5)	339 (8.3)	176 (8.9)	438 (13.0)	77 (2.2)	382 (9.9)	102 (3.3)	1,710 (7.6)
Maternal education (Illiterate)	2,299 (89.0)	2,767 (67.6)	532 (27.2)	44 (1.3)	913 (25.6)	472 (12.2)	2,157 (69.8)	9,184 (40.8)
Primary caretaker mother	68 (2.6)	306 (7.5)	145 (7.3)	803 (23.9)	913 (25.5)	1,047 (27.1)	207 (6.7)	3,489 (15.5)
Under five children in the house	5.83 (3.9)	3.37 (2.4)	1.92 (1.0)	1.79 (0.7)	1.47 (0.7)	1.35 (0.6)	2.26 (1.2)	2.49 (2.3)
Predominant floor (earth/sand)	301 (11.6)	59 (1.4)	309 (15.6)	1,029 (30.6)	159 (4.4)	3,135 (81.2)	785 (25.4)	5,777 (25.6)
Presence of animal at household	1,010 (98.2)	1,770 (87.1)	561 (82.3)	1,468 (99.5)	1,557 (99.3)	1,355 (97.2)	377 (29.9)	8,098 (85.8)
WASH								
Main source of drinking water (tube well)	524 (20.2)	2 (0.1)	107 (5.4)	174 (5.2)	23 (0.6)	3,839 (99.5)	0 (0)	4,669 (20.7)
Improved toilet facility	2,586 (99.5)	4,094 (99.9)	1,942 (98.2)	2,535 (75.5)	3,524 (98.4)	3,577 (92.7)	3,010 (97.2)	21,268 (94.2)
Handwash with water and soap	1,997 (76.9)	3,112 (75.9)	1,388 (70.3)	3,113 (92.7)	1,974 (55.1)	3,263 (84.6)	2,046 (66.1)	16,893 (74.7)
Practice handwashing								
Before nursing a child	1,039 (39.9)	1,015 (24.8)	1,106 (55.9)	867 (25.8)	2,717 (75.9)	913 (23.7)	1,159 (37.4)	8,816 (39.1)
After cleaning the child	2,102 (80.9)	1,729 (42.2)	862 (43.6)	703 (20.9)	2,067 (57.7)	1,382 (35.8)	1,593 (51.5)	10,438 (46.3)

GEMS = Global Enteric Multicenter Study; HAZ = height-for-age *z*-score; MUAC = in cm (mean; for children < 5 years of age) mid-upper arm circumference; diarrhea = three or more stool/day; fever = measured at least 38°C; WASH = water, sanitation, and hygiene; WAZ = weight-for-age *z*-score; WHZ = weight-for-height *z*-score. Maternal education (illiterate: who never go to school/have no academic education). Main source of drinking water categorized as tube well (shallow and deep tube well) and nontube well (piped into house/yard, public tap, open well, pond/lake, covered well, spring, river/rain water, bought, and bore hole and others).

* Mean ± SD.

**Figure 2.**
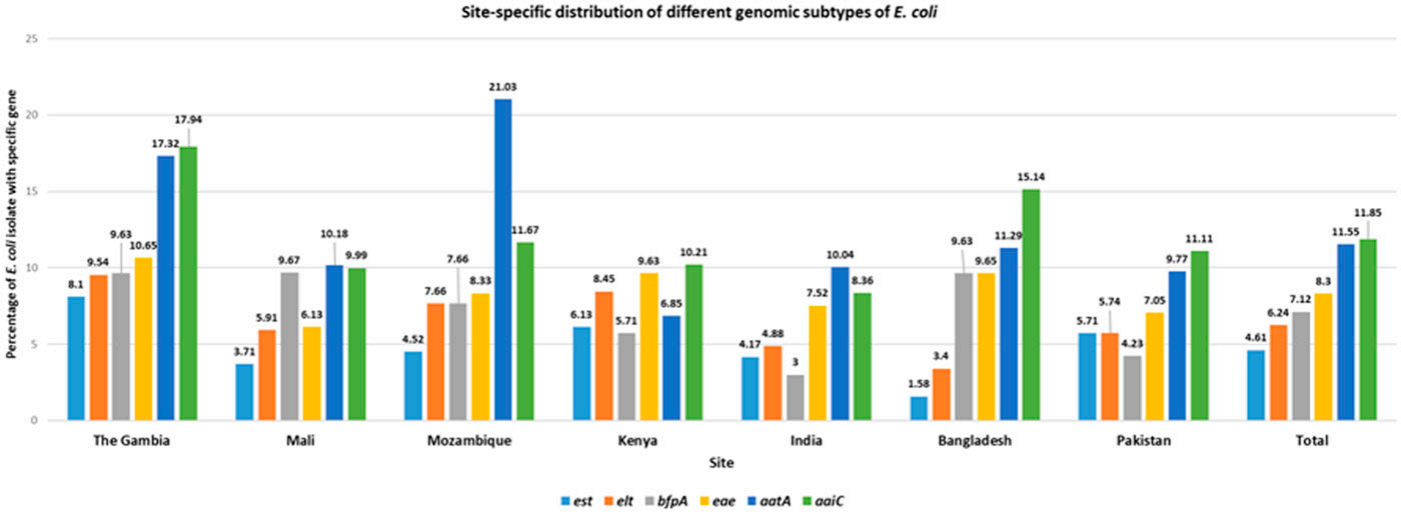
Site-specific distribution of different genomic subtypes of *E. coli.*

The different pathogenic variants of *E. coli* in the stool samples collected across all the seven study sites have been shown in Figure 2. Overall detection of *aaiC* gene of EAEC was most commonly detected (12%) and was highest in The Gambia (18%). In Mozambique, *aatA* gene of EAEC was found to be the highest (21.03%). It was also observed that *est* gene of ETEC was lowest among almost all the countries except Kenya, India, and Pakistan. In Bangladesh, *est* gene of ETEC was the lowest (1.58%). The *bfpA* gene of EPEC was found to be the lowest in India (3%) (Figure 2).

### Association between the different genomic variants of *E. coli* and child growth.

**Figure 3.**
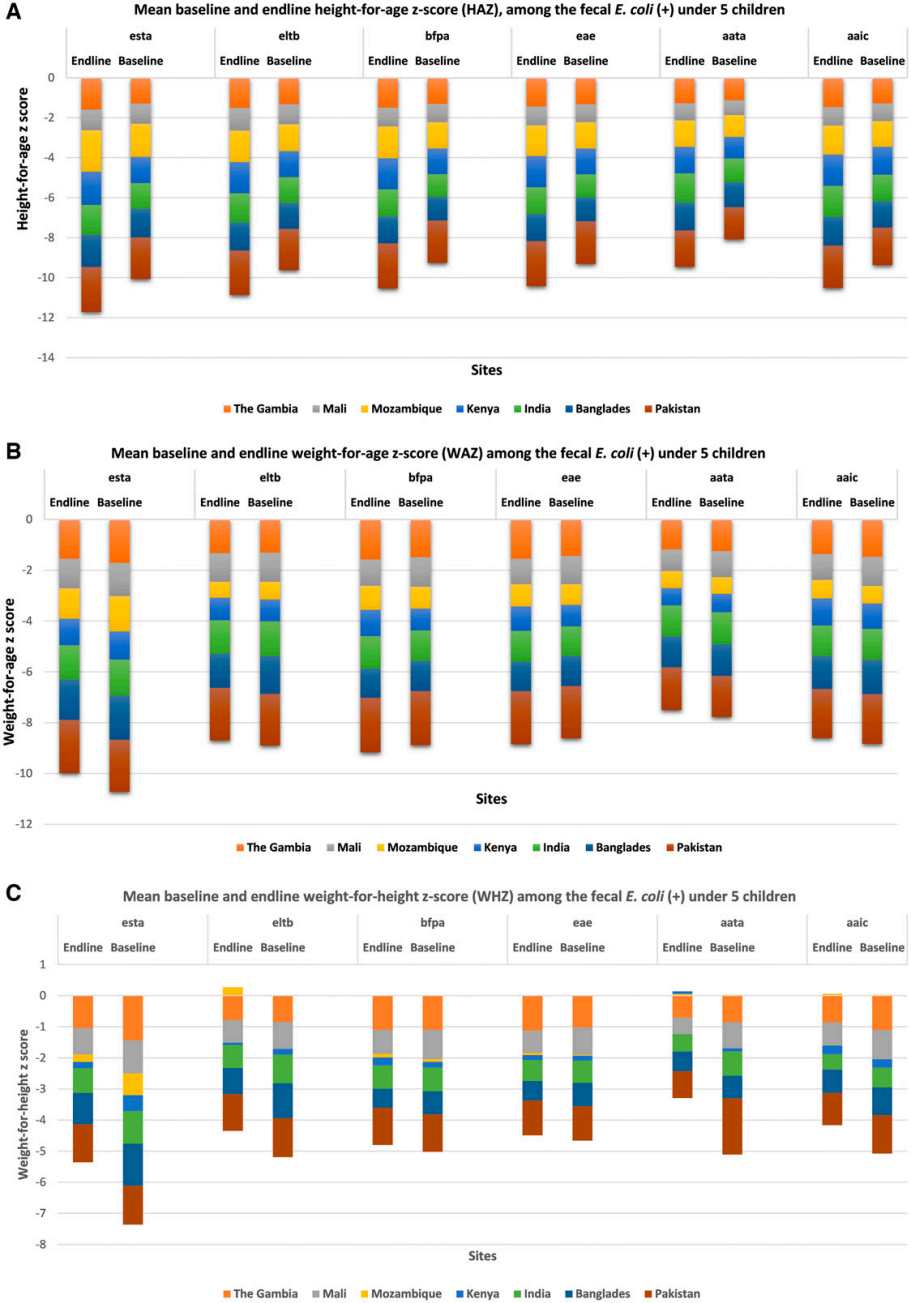
Mean baseline and endline height-for-age *z*-score (HAZ), weight-for-age *z*-score (WAZ), and weight-for-height *z*-score (WHZ) among the fecal *E. coli* (+) under 5 children.

Among the different pathogenic variants of *E. coli*-positive children under the age of 5, the mean HAZ score was found to be worsened at the endline, but mean WAZ and WHZ scores improved in the endline except for that in the case of *bfpA* and *eae* genes (Figure 3).

The outcomes of the multiple linear regression model are presented in Table 2. An important correlation between pathogenic variants of *E. coli* with WAZ and WHZ (*P* < 0.05) at the endline is shown in the unadjusted model. After adjusting for age, gender, diarrhea, breastfeeding status, mother’s education, number of under-5 children in the house, handwashing before nursing a child and after cleaning the child, type of handwashing material, the main source of drinking water, wealth index, available toilet facility, copathogens (*Campylobacter* and *Giardia*), comorbidity (malaria, typhoid, pneumonia, diarrhea, and dysentery), time (anthropometry at enrollment and on day 60 follow-up), and study site, in the multivariable model, a negative association of WAZ score and WHZ score with pathogenic variants of *E.coli*, namely: *est, bfpA, aatA, *and *aaiC* were found (Table 2); *est* virulence-related genes of ETEC were negatively associated with HAZ; and *aatA* pathogenic variant of EAEC were positively associated with HAZ, WAZ, and WHZ (Table 2).

**Table 2:** Association of pathogenic variants of *E. coli* infection with child’s HAZ, WAZ, and WHZ: results of multiple linear regression modeling (dependent variables—HAZ, WAZ, and WHZ)

		Unadjusted		Adjusted*		
		Coef. (95% CI)	*P* value	Coef. (95% CI)	*P* value	Effect size
ETEC	*est*					
	HAZ	–0.15 (–0.24, –0.08)	< 0.001	–0.09 (–0.14, –0.03)	0.004	–0.013
	WAZ	–0.29 (–0.39, –0.23)	–0.001	–0.24 (–0.30, –0.18)	< 0.001	–0.037
	WHZ	–0.32 (–0.41, –0.29)	< 0.001	–0.29 (–0.38, –0.21)	< 0.001	–0.042
	*Elt*					
	HAZ	–0.04 (–0.11, 0.03)	0.142	–0.04 (–0.10, 0.04)	0.161	–0.007
	WAZ	–0.02 (–0.09, 0.04)	0.475	–0.04 (–0.11, 0.03)	0.091	–0.008
	WHZ	–0.02 (–0.09, 0.05)	0.556	–0.05 (–0.11, 0.03)	0.111	–0.008
EPEC	*bfpA*					
	HAZ	0.08 (0.01, 0.13)	0.001	–0.03 (−0.11, 0.02)	0.153	–0.007
	WAZ	–0.05 (–0.13, 0.004)	0.074	–0.09 (–0.17, –0.04)	< 0.001	–0.018
	WHZ	–0.12 (–1.9, –0.07)	< 0.001	–0.10 (–0.18, –0.04)	< 0.001	–0.019
	*Eae*					
	HAZ	0.01 (–0.06, 0.065)	0.653	–0.02 (–0.08, 0.03)	0.443	–0.004
	WAZ	−0.03 (–0.11, 0.02)	0.154	–0.06 (–0.13, –0.01)	0.016	–0.011
	WHZ	–0.05 (–0.12, 0.004)	0.049	–0.06 (–0.13, –0.01)	0.010	–0.012
EAEC	*aatA*					
	HAZ	0.19 (0.14, 0.24)	< 0.001	0.06 (0.004, 0.11)	0.005	0.013
	WAZ	0.15 (0.10, 0.21)	< 0.001	0.06 (0.01, 0.12)	0.003	0.014
	WHZ	0.11 (0.07, 0.17)	< 0.001	0.06 (0.003, 0.11)	0.012	0.012
	*aaiC*					
	HAZ	0.001 (−0.050, 0.053)	0.973	–0.02 (–0.08, 0.02)	0.205	–0.006
	WAZ	−0.08 (−0.14, −0.03)	0.001	–0.07 (–0.12, –0.02)	0.001	–0.016
	WHZ	−0.11 (−0.17, −0.07)	< 0.001	–0.08 (–0.13, –0.03)	< 0.001	–0.017

Coef. = coefficient; EAEC = enteroaggregative *E. coli*; EPEC = enteropathogenic *E. coli*; ETEC = enterotoxigenic *E. coli*; HAZ = height-for-age; WAZ = weight-for-age; WHZ = weight-for-height *z*-scores. *Escherichia coli* was detected from the stool sample during enrollment. Anthropometric measurements were taken during enrollment and after 60 days of enrollment (during the follow-up visit). Separate models were performed to see the association of pathogenic variants of *E. coli* infection with child’s height-for-age, weight-for-age, and weight-for-height *z*-scores for the different genomic variants of *E. coli.*

* Adjusted for age, gender, diarrhea, breastfeeding status, mother’s education, number of children under the age of 5 in the house, handwashing before nursing a child and after cleaning the child, handwashing material, main source of drinking water, available toilet facility, wealth index, copathogens (*Campylobacte*r and *Giardia)*, site, and comorbidity (malaria, typhoid, pneumonia, diarrhea, and dysentery).

In Table 3, we showed the association of pathogenic variants of *E. coli* with child growth in different age group, where after adjusting for all potential covariates we found a negative correlation between *est* subtype of diarrhoeagenic ETEC with WAZ [Coef. −0.14 (95% CI: −0.28, 0.002); *P* value = 0.046], WAZ [Coef. −0.14 (95% CI: −0.24, −0.04); *P* value = 0.008] and, WHZ [Coef. −0.11 (95% CI: 0.22, −0.02); *P* value = 0.034] among 24–60 months age children. But among 0–23 months of age of children, we observed a negative association between *est* subtype of ETEC with HAZ, WAZ, and WHZ. There was no association between the other subtypes of *E. coli* with child growth among the older children who were aged 24–60 months. And among the younger children (0–23 months) the *bfpa* and *aaiC* virulence genes were negatively associated with WAZ and WHZ. There was also a negative association between *eta* variant of ETEC and HAZ who were from the 0–23 months age group. But *aatA* had a positive association with child growth among young children.

**Table 3:** Association of pathogenic variants of *E. coli* infection with child’s HAZ, WAZ, and WHZ: results of multiple linear regression modeling (dependent variables—HAZ, WAZ, and WHZ) among different age group

		0–23 months			24–60 months		
Age group		Coef. (95% CI) *	*P* value	Effect size	Coef. (95% CI) *	*P* value	Effect size
ETEC	*est*						
	HAZ	–0.10 (–0.17, –0.03)	0.007	–0.015	–0.13 (0.24, –0.02)	0.024	–0.020
	WAZ	–0.28 (0.36, –0.21)	< 0.001	–0.043	–0.14 (−0.24, –0.04)	0.008	–0.023
	WHZ	–0.35 (–0.43, –0.27)	< 0.001	–0.049	–0.11 (0.22, –0.01)	0.034	–0.019
	*elt*						
	HAZ	–0.07 (–0.12, –0.01)	0.026	–0.012	–0.02 (–0.12, 0.08)	0.693	–0.004
	WAZ	–0.04 (–0.11, 0.02)	0.152	–0.008	–0.08 (–0.17, 0.02)	0.103	–0.014
	WHZ	–0.04 (–0.1, 0.03)	0.286	–0.006	–0.10 (–0.19, 0.001)	0.050	–0.017
EPEC	*bfpA*						
	HAZ	–0.03 (–0.09, 0.02)	0.256	–0.006	0.02 (–0.08, 0.12)	0.716	0.003
	WAZ	–0.11 (–0.17, −0.05)	< 0.001	–0.021	–0.01 (–0.1, 0.08)	0.791	–0.002
	WHZ	–0.12 (–0.18, −0.06)	< 0.001	–0.021	–0.03 (–0.12, 0.06)	0.505	–0.006
	*eae*						
	HAZ	–0.01 (–0.06, 0.04)	0.662	–0.002	0.02 (–0.07, 0.11)	0.616	0.004
	WAZ	–0.05 (–0.11, 0.001)	0.046	–0.011	–0.04 (–0.12, 0.05)	0.392	–0.008
	WHZ	–0.06 (–0.12, 0.001)	0.051	–0.011	–0.06 (–0.15, 0.02)	0.140	–0.013
EAEC	*aatA*						
	HAZ	0.11 (0.07, 0.16)	< 0.001	0.030	0.03 (–0.07, 0.12)	0.613	0.005
	WAZ	0.08 (0.03, 0.12)	0.001	0.019	0.06 (–0.03, 0.15)	0.175	0.012
	WHZ	0.07 (0.02, 0.12)	0.005	0.016	0.06 (–0.03, 0.15)	0.199	0.011
	*aaiC*						
	HAZ	–0.03 (–0.07, 0.02)	0.217	–0.007	–0.01 (−0.09, 0.06)	0.746	–0.003
	WAZ	–0.09 (–0.13, –0.04)	< 0.001	–0.021	0.01 (–0.06, 0.07)	0.870	0.001
	WHZ	–0.10 (–0.15, –0.05)	–0.001	–0.022	0.01 (–0.06, 0.08)	0.759	0.003

Coef. = coefficient; EAEC = enteroaggregative *E. coli*; EPEC = enteropathogenic *E. coli*; ETEC = enterotoxigenic *E. coli*; HAZ = height-for-age; WAZ = weight-for-age; WHZ = weight-for-height *z*-scores. *Escherichia coli* was detected from the stool sample during enrollment. Anthropometric measurements were taken during enrollment and after 60 days of enrollment (during the follow-up visit). Separate models were performed to see the association of pathogenic variants of *E. coli* infection with child’s height-for-age, weight-for-age, and weight-for-height *z*-scores for the different genomic variants of *E. coli.*

* Adjusted for age, gender, diarrhea, breastfeeding status, mother’s education, number of children under the age of 5 in the house, handwashing before nursing a child and after cleaning the child, handwashing material, main source of drinking water, available toilet facility, wealth index, copathogens (*Campylobacte*r and *Giardia)*, site, and comorbidity (malaria, typhoid, pneumonia, diarrhea, and dysentery).

Only in Bangladesh, *elt gene* of ETEC was significantly associated with poor WHZ [coef. −0.21 (95% CI: −0.39, −0.02), *P* value: 0.026] after adjusting for the potential covariates. On the other hand, *est* pathogenic variant of ETEC was negatively associated with child HAZ score in Mozambique [coef. −0.34 (95% CI: −0.65, −0.03), *P* value: 0.030]. In Pakistan, *aatA* pathogenic variant of EAEC was positively associated with WAZ [coef. 0.22 (95% CI: 0.05, 0.38), *P* value: 0.012], WHZ [coef. 0.18 (95% CI: 0.02, 0.35), *P* value: 0.032], and HAZ [coef. 0.18 (95% CI: 0.01, 0.35), *P* value: 0.039] (Supplemental Table 1).

## DISCUSSION

To our knowledge, this is the first study to look into the link between distinct pathogenic variants of *E. coli* and growth in under-5 children irrespective of diarrhea. The GEMS previously showed a 38% incidence rate for only the *aaiC* pathogenic variant among under-5 MSD children.^39^

In our study, we found a positive association of *aaiC* pathogenic variant of EAEC with childhood malnutrition (wasted and underweight), but the association observed between *aaiC* with child linear growth was not statistically significant. The relationship between *Shigella* and ETEC-related illness, weight gain, and linear growth in Bangladeshi children aged 0–5 years was investigated by Black et al. They found diarrhea associated with ETEC had a significant negative effect on the bimonthly weight gain of children in rural community.^40^ Another study conducted among 0- to 6-year-old children in the Peruvian Amazon region predicted similar relationships^41^ and found that the association between ETEC diarrhea and weight gain was twice that of other etiologic agents.^41^ A multicountry cohort study, MAL-ED, found that EAEC had an inverse association with linear growth at 24 months of age.^16^ The increased prevalence of the *aaiC* pathogenic variant in our study compared with other EAEC variants may provide more evidence for the plasmid aggregative adherence (pAA’s) gene transfer. Our findings show that *aaiC* is an incongruent marker for EAEC identification and our findings are consistent with those of research conducted in southern Mozambique.^42^ However, we do not have any conclusive explanation for the favorable correlation between EAEC’s *aatA* pathogenic variant and child growth in Pakistan and warrants further meticulous investigation. However, in the MAL-ED study, the presence of EAEC variants harboring both *aaiC* and *aatA* was linked to a higher risk of growth failure in Bangladeshi children.^16^ Other studies suggested that the presence of different virulence genes from within and outside the plasmid AA is necessary for complete EAEC virulence.^43^ In the case of EAEC, malnutrition and growth impairment may be due to intestinal inflammation or to the thick mucosal gel with which they are associated in human intestinal explants, which could theoretically impair absorption of nutrients.^44^

The presence of both *bfpA* and *eae* pathogenic variants of EPEC was also found to be negatively associated with child growth in our analysis in the current study. In Norwegian children, a substantial link was found between aEPEC (*eae* gene) and diarrhea lasting 14 days or more, suggesting that aEPEC may play a role in chronic illness^45^ and which might cause chronic malnutrition. Attaching and effacement (A/E) lesions have occurred when various genetic subgroups of EPEC adhere to gut epithelial cells in vitro or in vivo. The majority of children with EPEC infections were found to have anomalies such as a decrease in the number and height of microvilli, blunting of enterocyte boundaries, loss of the glycocalyx, shortening of villi, and the appearance of a mucus pseudomembrane on the mucosal surface.^46^ These ultrastructural changes could be the result of a system in which distinct EPEC subgroups produce diarrhea in response to food or water contamination, which, if persistent, can lead to chronic malnutrition in children.

Despite a wealth of studies revealing disease processes, immunological responses, and vaccine development, ETEC remains poorly understood in terms of mediation of virulence and pathogenesis and the precise immune responses mounted by the host system.^47^ In our study, we noticed that the pathogenic variant of ETEC was negatively linked with child growth. When EPEC variants adhere to epithelial cells in vitro or in vivo, they generate attaching and effacement (A/E) lesions.^46^ Although nonspecific, surface abnormalities of the small intestine mucosa as evidenced by scanning electron microscopy in neonates with chronic diarrhea are severe enough to justify the severity of the clinical characteristics seen at such a young age. The majority of patients had anomalies such as a decrease in the number and height of microvilli, blunting of enterocyte boundaries, loss of the glycocalyx, shortening of villi, and the appearance of a mucus pseudomembrane on the mucosal surface.^46^ These ultrastructural changes could be the result of a link between the enteric enteropathogenic agent that causes diarrhea and the emergence of food intolerance, which leads to diarrhea and malnutrition.

Data analysis from the WHO Global Database on Child Growth and Malnutrition from 57 countries found weight for height starts slightly above the standard in children aged 1 to 2 months and falters slightly until 9 months of age, picking up after that age and remaining close to the standard thereafter. Weight for age starts close to the standard and falters moderately until reaching 24 months and remaining reasonably stable after that.^48^ In our study, we observed that only *est* genomic variant of ETEC had a negative association with child nutrition after 24 months of age, which was consistent with other studies. There was a negative association with HAZ between *elt* and *aatA* genomic variants of *E. coli* in our study only in the younger age group, but not among the older children. Another study from rural Pakistan observed that the children who were stunted at 18 months remain stunted at 48 months.^49^ Most studies have shown that major linear growth failure occurs in the first 48 months of life; beyond this age, catch-up growth is rare.^50^^,^^51^ Further studies will be required to evaluate this type of findings among older children.

Our study’s strengths included unbiased random sampling, a large sample size, and high-quality laboratory performance. However, in all sites, cost-free healthcare is provided to all persons, regardless of their socioeconomic or other contexts, increasing the likelihood of more people from poor socioeconomic backgrounds enrolling as study participants. We investigated for a link between symptomatic and asymptomatic *E. coli* infection and growth stalling in children under the age of 5 at seven different sites, which enriched the study’s findings. A notable feature of this study was the single follow-up household visit, roughly 60 days after enrollment, consequently, the observed association may be due to an acute effect. Moreover, it is possible that the effect of nutrition status at baseline influenced *E. coli* variant infection. The impact of the use of antibiotics before recruitment may influence our study findings, it was another limitation of our study. The quantitative approach, while incrementally useful, will inherently function less well for pathogens that are shed with high frequency, in high quantities, and for an extended duration in the absence of diarrhea. Finally, vaccine development often relies on speciation and subtyping of infections, and the molecular assays did not provide such information.

## CONCLUSION

In conclusion, we revealed a negative relationship between the pathogenic variants of *E. coli*, namely: *est, bfpA, eae*, and *aaiC* with WAZ and WHZ, as well as a positive association between *aatA* pathogenic variant with WAZ, WHZ, and HAZ. These findings point to the need for preventive methods and vaccine development aimed at distinct pathogenic variants of *E. coli*, which could help to minimize disease burden and its consequences, such as stunting in children’s growth during their first 5 years of life. As a result, a proven effective intervention, improvements in household water, sanitation, and hygiene practices, and efficient treatment of *E. coli* gastroenteritis are all urgently needed to lessen the adverse effects of undernutrition in such children.

## Supplemental Material


Supplemental materials
