# Supplementary material for: Association between Pathogenic Variants of Diarrheagenic Escherichia coli and Growth in Children under 5 Years of Age in the Global Enteric Multicenter Study
Source: Am J Trop Med Hyg. 2022 Jun 6;107(1):72–81. doi: 10.4269/ajtmh.22-0096 (PMC9294710; doi:10.4269/ajtmh.22-0096)
Supplement: Supplementary file 1 [file tpmd220096.SD1.pdf]

Supplemental Table 1. Site-specific association of pathogenic variants of *E. coli* infection with the child's HAZ, WAZ, and WHZ: results of multiple linear regression modeling (dependent variables—HAZ, WAZ, and WHZ)

	**ETEC**				**EPEC**				**EAEC**			
	est		***elt***		***bfpA***		***eae***		***aatA***		***aaiC***	
**Site**	**Coef. (95%CI)**	**P value**	**Coef. (95%CI)**	**P value**	**Coef. (95%CI)**	**P value**	**Coef. (95%CI)**	**P value**	**Coef. (95%CI)**	**P value**	**Coef. (95%CI)**	**P value**
						**HAZ**						
**The Gambia**	-0.17(-0.37,0.02)	0.081	-0.11(-0.29,0.07)	0.243	-0.17 (-0.34,0.01)	0.065	-0.13 (-0.30, 0.04)	0.128	0.06 (-0.081, 0.20)	0.403	-0.12 (-0.25, 0.02)	0.094
**Mali**	-0.03(-0.24,0.17)	0.750	-0.15(-0.31,0.01)	0.071	-0.01 (-0.14,0.13)	0.938	0.001 (-0.16, 0.16)	0.990	0.07 (-0.05, 0.20)	0.259	0.03 (-0.10, 0.16)	0.644
**Mozambique**	-0.34(-0.65, -0.03)	0.030	0.05(-0.18,0.28)	0.650	-0.09 (-0.32,0.14)	0.449	-0.07 (-0.29, 0.15)	0.560	0.13 (-0.02, 0.29)	0.079	0.10 (-0.09, 0.28)	0.307
**Kenya**	-0.08(-0.25,0.09)	0.334	0.04(-0.1,0.19)	0.577	-0.14 (-0.31,0.03)	0.117	-0.08 (-0.22, 0.05)	0.228	0.001 (-0.16, 0.16)	0.987	0.09 (-0.22, 0.05)	0.201
**India**	0.08(-0.1,0.26)	0.382	0.09(-0.08,0.26)	0.304	0.22 (0.01, 0.44)	0.051	0.18 (0.04, 0.32)	0.012	0.05 (-0.07, 0.18)	0.418	-0.08 (-0.21, 0.05)	0.244
**Bangladesh**	-0.12(-0.39,0.15)	0.395	0.01(-0.18,0.19)	0.957	0.05 (-0.07,0.16)	0.399	0.04 (-0.07, 0.15)	0.497	-0.03 (-0.14, 0.08)	0.607	-0.01 (-0.10,0.09)	0.911
**Pakistan**	-0.14(-0.37,0.09)	0.222	-0.11(-0.33,0.12)	0.346	-0.28 (-0.53, -0.03)	0.029	-0.22 (-0.41, -0.02)	0.030	0.18 (0.01, 0.35)	0.039	-0.01 (-0.18, 0.15)	0.878
						**WAZ**						
**The Gambia**	-0.32(-0.52, -0.13)	0.001	0.06(-0.12,0.24)	0.506	-0.26 (-0.44, -0.08)	0.005	-0.21 (-0.38, -0.03)	0.019	0.13(0.01,0.28)	0.069	-0.15 (-0.28, -0.01)	0.035
**Mali**	-0.15(-0.35,0.05)	0.142	-0.08(-0.24,0.07)	0.296	-0.07 (-0.2,0.05)	0.253	-0.01 (-0.17, 0.14)	0.860	0.10 (-0.03, 0.22)	0.119	0.06 (-0.18, 0.07)	0.372
**Mozambique**	-0.59(-0.9, -0.28)	<0.001	0.08(-0.15,0.31)	0.486	-0.33 (-0.56, -0.1)	0.005	-0.29 (-0.51, -0.07)	0.010	-0.03 (-0.18, 0.13)	0.746	0.03 (-0.16, 0.21)	0.788
**Kenya**	-0.26(-0.43, -0.08)	0.004	-0.01(-0.16,0.14)	0.923	-0.19 (-0.37, -0.01)	0.035	-0.12 (-0.26, 0.03)	0.108	0.08 (-0.09, 0.25)	0.356	-0.20 (-0.34, -0.07)	0.003
**India**	-0.09(-0.28,0.1)	0.372	-0.04(-0.22,0.14)	0.648	0.06 (-0.16,0.29)	0.587	0.09 (-0.06, 0.24)	0.232	-0.01 (-0.14, 0.12)	0.882	0.01 (-0.13, 0.15)	0.886
**Bangladesh**	-0.29(-0.56, -0.02)	0.036	-0.15(-0.33,0.04)	0.127	0.09 (-0.03,0.2)	0.131	0.07 (-0.04, 0.19)	0.212	0.002 (-0.11, 0.11)	0.972	-0.01 (-0.10, 0.09)	0.861
**Pakistan**	-0.2(-0.42,0.02)	0.082	-0.15(-0.37,0.06)	0.167	-0.35 (-0.6, -0.11)	0.005	-0.23 (-0.43, -0.04)	0.016	0.22 (0.05, 0.38)	0.012	-0.11 (-0.28, 0.05)	0.166
						**WHZ**						
**The Gambia**	-0.35(-0.56, -0.14)	0.001	0.15(-0.05, 0.34)	0.144	-0.23 (-0.42, -0.04)	0.020	-0.19 (-0.38, -0.01)	0.042	0.17 (0.01, 0.32)	0.035	0.13 (-0.28, 0.02)	0.080
**Mali**	-0.18(-0.4, 0.04)	0.107	-0.02(-0.19, 0.15)	0.818	-0.11 (-0.25,0.03)	0.120	-0.03 (-0.20, 0.13)	0.692	0.09 (-0.05, 0.22)	0.201	0.11 (-0.24, 0.03)	0.121
**Mozambique**	-0.64(-0.98, -0.31)	<0.001	0.04(-0.21, 0.29)	0.776	-0.35 (-0.6, -0.1)	0.005	-0.31 (-0.55, -0.08)	0.010	0.15 (-0.31, 0.02)	0.082	-0.06 (-0.26, 0.14)	0.565
**Kenya**	-0.28(-0.45, -0.11)	0.001	-0.03(-0.18, 0.11)	0.643	-0.16 (-0.33,0.02)	0.074	-0.09 (-0.23, 0.04)	0.184	0.12 (-0.04, 0.28)	0.155	-0.19 (-0.32, -0.06)	0.005
**India**	-0.2(-0.39,0)	0.046	-0.14(-0.32, 0.05)	0.143	-0.07 (-0.31,0.16)	0.545	-0.02 (-0.17, 0.13)	0.792	-0.05 (-0.18, 0.09)	0.486	0.08 (-0.06, 0.22)	0.264
**Bangladesh**	-0.31(-0.58, -0.05)	0.021	-0.21(-0.39, -0.02)	0.026	0.07 (-0.04,0.18)	0.203	0.05 (-0.06, 0.17)	0.342	0.04 (-0.06, 0.15)	0.440	-0.02 (-0.11, 0.07)	0.688
**Pakistan**	-0.19(-0.41,0.03)	0.091	-0.14(-0.36,0.07)	0.198	-0.23 (-0.47,0.02)	0.070	-0.12 (-0.31, 0.07)	0.215	0.18 (0.02, 0.35)	0.032	-0.14 (-0.30, 0.02)	0.093

* adjusted for age, gender, diarrhea, breastfeeding status, mother's education, number of children under the age of 5 in the house, handwashing before nursing a child and after cleaning the child, handwashing material, main source of drinking water, available toilet facility, wealth index, co-pathogens (*Campylobacter* and *Giardia)*, and comorbidity (malaria, typhoid, pneumonia, diarrhea, dysentery). Abbreviation: Coef.: coefficient, CI: confidence interval; HAZ: height-for-age, WAZ: weight-for-age, and WHZ: weight-for-height z-scores*; E. coli* was detected from the stool sample during enrollment; Anthropometric measurements were taken during enrollment and after 60 days of enrollment (during the follow up visit)
